# The Use of a Novel Term Helps Preschoolers Learn the Concept of Angle: An Intervention Study With Chinese Preschool Children

**DOI:** 10.3389/fpsyg.2020.568388

**Published:** 2020-11-20

**Authors:** Xiaohui Xu, Chuansheng Chen, Jianfang Ma, Xiaoting Zhao, Mengwen Jiao, Zhiyong Xin

**Affiliations:** ^1^School of Preschool Education, Capital Normal University, Beijing, China; ^2^Department of Psychological Science, University of California, Irvine, Irvine, CA, United States; ^3^School of Sociology and Psychology, Central University of Finance and Economics, Beijing, China

**Keywords:** preschooler, angle, intervention, novel term, whole-object assumption

## Abstract

Angle is an important concept in geometry. Young children have difficulty separating angle size from other dimensions such as the length of angle sides, perhaps due to whole-object bias in word learning. The present study used the pre-test–training–post-test design to investigate the effectiveness of two ways of separating angle from angle size in 3–6-year-old Chinese preschoolers. A total of 228 children were given a pre-test and 219 of them failed the crucial test. 168 of the 219 children were present at school during the training phase and were randomly assigned to three groups: the “toma” group (*n* = 57), which received training to call the whole angle figure as “toma” and angle size as angle size; the “angle/angle size” group (*n* = 56), which received the training of separating “angle” from “angle size”; and the control group (*n* = 55), which used “angle size” alone to represent both the overall angle figure and angle size. Results showed that the “toma” group improved significantly more than the other two groups, the latter of which did not differ from each other. These results suggest that it is insufficient to have two separate words/phrases (angle and angle size) for children to learn to differentiate angle from angle size, perhaps due to their shared usage of the word *angle*. Instead, the use of a novel term is necessary and sufficient to improve learning. Implications for preschool education are discussed.

## Introduction

Angles are an important visual experience. As early as a few hours after birth, neonates already show sensitivity to the two fundamental properties of Euclidean geometry, angle and length (Schwartz et al., 1979; Cohen and Younger, 1984; Slater et al., 1991; Lourenco and Huttenlocher, 2008; Lindskog et al., 2019). Despite the early-developing sensitivity to angle, however, school children have great difficulty learning the concept of “angle size” (Clements and Battista, 1992; Mitchelmore and White, 2000). When comparing the size of angles, elementary and even middle school students are often confused by irrelevant properties such as the length of an angle’s sides, the area within the sides, and the distance between the sides. For example, they would mistakenly judge that the angle formed by longer lines is larger than the same angle formed by shorter lines (Clements and Battista, 1989; Lindquist and Kouba, 1989; Lehrer et al., 1998; Huangpu, 2009).

Why do school children fail to take advantage of early sensitivity to angle to learn the concept of angle size? According to Van Hiele’s model of geometric reasoning (Van Hiele, 1986), children learn to use a holistic processing approach to understanding geometric shapes. When learning a new concept such as angle, children make the whole-object assumption, i.e., a novel label is likely to refer to the whole object and not to its parts, substance, or other properties (e.g., Markman and Hutchinson, 1984; Landau et al., 1988; Hollich et al., 2007). When an adult points to an angle size and says “angle or angle size” in the classroom or in daily life, children map “angle size” to the entire angle figure. Therefore, they do not separate the size of an angle from its other dimensions such as length or area (Clements and Battista, 1989). What can educators do to help children to learn the concept of angle size? One way to force the children to separate angle size from other dimensions is to give separate labels for the angle size (“angle”) and the whole angle figure (“toma”), as demonstrated by Gibson et al. (2015). Specifically, children in the experimental condition were given a label “toma” referring to the entire angle and “angle” referring to the size of an angle, and children in the control condition were given one label “angle” referring to both the whole angle and angle size as is done in daily English usage. Result showed that children in the experimental condition significantly improved in their understanding of the concept of angle size and the improvement was greater than that in the control condition.

Building on Gibson et al.’s (2015) finding that a novel term can help 4 year olds learn to differentiate angle size from angle, the current study aimed to expand this line of research in the following ways. First, we extended this research to a Chinese sample. Cultural differences in general and linguistic differences in particular between Chinese and Americans have been found to affect language learning (Tardif et al., 1997, 1999) and mathematical cognition even in preschoolers (e.g., Kelly et al., 1999; Zhou et al., 2007; Siegler and Mu, 2008; Xu et al., 2013). This study aimed to test whether the “toma” intervention would also be effective among a group of Chinese preschoolers. Second, in addition to the “toma” intervention condition and a control condition, we added a third condition that included another way of separating “angle (jiao)” and “angle size (jiaodu)” commonly used by Chinese elementary and middle school teachers. If the mere use of separate labels would be sufficient, the use of two labels “angle” and “angle size” should lead to improved learning of the concept of angle. On the other hand, the fact that both labels still include the word “angle” may not help children to learn the concept of angle. Third, previous studies have found significant gender differences in children’s geometric and spatial cognition (Spelke, 2005; Davies and Uttal, 2007; Halpern et al., 2007; Tzuriel and Egozi, 2010; Tian et al., 2018). Gender difference was not examined in Gibson et al.’s study, perhaps due to its limited sample size (*N* = 30). This study used a much larger sample (*N* = 228) and investigated gender differences in children’s understanding of angle and angle size and in the potential effects of the interventions. Fourth, age is another factor that was not examined in Gibson et al.’s study, which included only 4–5-year-olds (*M*_age_ = 4.86 years). Given the rapid changes in whole-object bias and mutual exclusivity bias in early childhood (Markman and Wachtel, 1988; Soja et al., 1991; Hall et al., 1993), we included a wider age range (from 3 to 6 years of age) to examine whether the effect of the “toma” intervention would be similar for children younger and older than those in Gibson et al. experiment.

## Materials and Methods

### Participants

This experiment was carried out at two preschools in an average neighborhood in Beijing. Children were initially tested to assess their understanding of the concept of angle. Those who did not understand the concept served as the sample for experiment. The pre-test included 228 children from three grade levels (young, middle, and older preschoolers). The young (first-year) preschoolers included 72 children (44 boys, 28 girls, *M*_age_ = 3.65 years, *SD* = 3.15, age range: 3.25–4.25 years), the middle (second-year) preschoolers included 75 children (43 boys, 32 girls, *M*_age_ = 4.66 years, *SD* = 3.70, age range: 4.00–5.25 years), and the older (third-year) preschoolers (equivalent to American kindergarteners) included 81 children (49 boys, 32 girls, *M*_age_ = 5.66 years, *SD* = 3.52, age range: 5.00–6.25 years). Participation was voluntary and neither children nor teachers received compensation. Parental consent was obtained before the experiment. The experimental protocol was approved by the IRB of Capital Normal University.

Based on the pre-test, almost all children (219 of 228) lacked an understanding of angle size as a separate dimension from the angle figure (see Table 1). The subjects who lacked an understanding of angle served as the pool for the intervention. Due to illness and other reasons (vacation, celebrations, etc.), 51 children did not come to school to take part in the experiment. The rest of the children (*N* = 168) finished the experiment. The “toma” group included 57 children (35 boys, 22 girls; 19 young preschoolers, 20 middle preschoolers, 18 older preschoolers), the “angle/angle size” group included 56 children (34 boys, 22 girls; 19 young preschoolers, 19 middle preschoolers, 18 older preschoolers), and the control group included 55 children (32 boys, 23 girls; 18 young preschoolers, 19 middle preschoolers, 18 older preschoolers).

**TABLE 1:** Distribution of subjects with different pre-test scores on the length-inconsistent task.

	0 points	Above 0 points	Total
Boy			
Young	44	0	44
Middle	43	0	43
Old	44	5	49
Total	131	5	136
Girl			
Young	28	0	28
Middle	30	2	32
Old	30	2	32
Total	88	4	92
Total			
Young	72	0	72
Middle	73	2	75
Old	74	7	81
Total	219	9	228

### The Conditions

For the “toma” condition, we used the Gibson intervention strategies. Children were taught that the label “toma (tuoma in Chinese)” represented the overall angle figure and the word “angle size (jiaodu)” referred to the measure of rotation of an angle figure (Note: Gibson et al. used “angle,” which can be translated into either jiao or jiaodu. We used “angle size (jiaodu)” because of its clarity in Chinese.). For the “angle/angle size” condition, children were taught that the label “angle (jiao)” represented the overall angle figure and the word “angle size (jiaodu)” referred to the measure of rotation of an angle figure, which was the same as in the “toma” condition. In the control condition, children only heard the word “angle size” in reference to both the overall angle figure and the measure of rotation of an angle figure. We could have used “angle (jiao)” in reference to both concepts for the control condition or for an additional condition. However, the ultimate aim is to help children learn the concept of angle size (jiaodu), so we used this term because of its precision and clarity (see Table 2).

**TABLE 2:** The terms used in Gibson et al.’s and current studies.

Condition	Gibson et al.’s study		Current study	
	Overall angle figure	Measure of rotation of an angle	Overall angle figure	Measure of rotation of an angle
Toma	Toma	Angle	Toma (tuoma)	Angle size (jiaodu)
Angle/angle size	–	–	Angle (jiao)	Angle size (jiaodu)
Control	Angle	Angle	Angle size (jiaodu)	Angle size (jiaodu)

### Pre-test and Post-test Task

**FIGURE 1:**
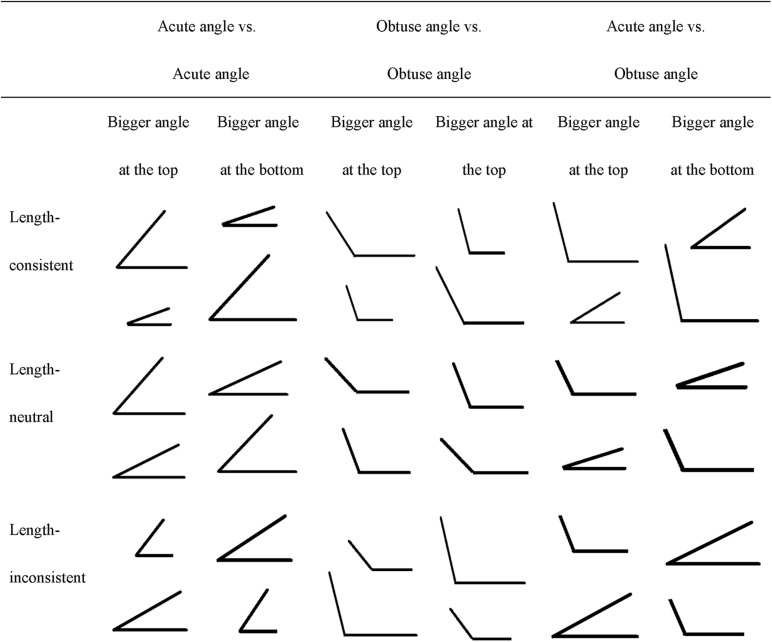
Angle comparison task.

We adapted Gibson et al.’s (2015) angle rotation comparison tasks. There were three types of angle rotation comparison tasks: *length-consistent*, *length-neutral*, and *length-inconsistent* trials, with 6 trials for each type and 18 trials in total (see Figure 1). On each trial, children were presented with a card depicting two angle figures and asked: “Can you tell me which one has the bigger angle size (jiaodu)?” Each angle figure was formed by two line segments that met at a single point. On the length-consistent trials, the larger angle size also had longer sides, and the smaller angle size had shorter sides. Children could judge correctly based on the length of the sides of the angles even if they were unable to properly compare the measurement of rotation of an angle figure. In other words, this task only assessed whether children had the general sense of larger or small geometric objects. On the length-neutral trials, the figure varied in angle size but not in length of the sides. This task controlled for one dimension (length) but no other dimensions (e.g., area), so it required a bit more sense of geometric shapes than the length-consistent task. On the length-inconsistent trials, the larger angle size had shorter sides than the smaller angle size. This is the crucial task that taps children’s true understanding of the dimension of angle size.

All figures were arranged in the same orientation with a horizontal base and the vertex on the left side of the page. There were six pairs of angle figures (two pairs of acute angles, two pairs of obtuse angles, and two pairs of one acute angle vs. one obtuse angle) in each type of tasks. The pair of angles for each trial were arranged vertically, one at the top and the other one at the bottom. Three pairs of angle figures had the bigger angles at the top, and the other three pairs had the bigger angles at the bottom. Children were given 1 point for each correct answer and 0 points for wrong answers. Each type of tasks had a score range of 0–6 points.

### Training

The training session consisted of three parts: introduction, description, and guided practice. The instructions were the same as those used by Gibson et al. (2015) except that they were given in Chinese.

In the introduction phase of training, all children were shown a picture of a single acute angle figure and then four pairs of angle figures (two pairs of acute angles and two pairs of obtuse angles). The angle figures within each pair had different side lengths but the same angle size. In the “toma” and “angle/angle size” experimental conditions, the experimenter pointed to the single acute figure and said, respectively, “This is a toma/angle. Can you say toma/angle.” Then in the “toma” experimental condition, the experimenter pointed to each figure and said: “Here are two tomas. Here is a bigger toma and here is a smaller toma. Can you point to the bigger toma? Can you point to the smaller toma?” In the “angle/angle size” experimental condition, the experimenter pointed to each figure and said: “Here are two angles. Here is a bigger angle and here is a smaller angle. Can you point to the bigger angle? Can you point to the smaller angle?” Each of the four trials was repeated once and the experimenter provided feedback regardless of whether the child was correct or incorrect (i.e., “Right! This is the bigger toma/angle!” or “Oops! This is the bigger toma/angle”). In the control condition, the experimenter pointed to the single acute angle and said: “This is an angle size. Can you say jiaodu?” Then experimenter simply pointed to each figure and said: “Here are two angle sizes. Here is an angle size and here is the other angle size. Can you point to an angle size? Can you point to the other angle size?” Each of the four trials was repeated once and the experimenter provided encouragement each time the child correctly pointed to the two angle sizes.

In the description phase of training, all children were shown a picture of a single angle figure with the arc of the angle highlighted by an arrow. The three groups of children were told, respectively: “Let’s take a close look at the toma/angle/angle size. There are two lines (experimenter traces the sides) the top line opens up (experimenter traces the arrow) to form an angle size (experimenter points to the center of the figure).” This was repeated three times for each child.

In the guided practice phase of training, all children were given three length-consistent and three length-inconsistent trials. The order of presentation of the trials was the same for every child (one pair of acute angles, one pair of obtuse angles, and one pair of one acute angle vs. one obtuse angle in each type of trails). In the two experimental conditions, children were presented with the first pair of angles and asked: “Can you point to the bigger toma/angle?” After the children answered, experimenter gave feedback to them (“Right! This is the bigger toma/angle” or “Oops! This is the bigger toma/angle”) and then told: “Now let’s look at the angle size. This is the bigger angle size (experimenter points to center of the figure with larger angle size) and this is the smaller angle size (experimenter points to center of the other figure). Can you show me the bigger angle size?” Again, children received feedback (Right! This is the bigger angle size” or “Oops! This is the bigger angle size”). This process was repeated for all six trials. After going through all trials once, the same six trials were repeated a second time during which the children were only asked: “Can you show me the bigger angle size?” Again, all children received feedback regardless of whether or not they were correct. In the control condition, children saw the same six trials, but were not asked to identify the larger toma/angle. They were only told: “Here are two angle sizes. This is the bigger angle size (experimenter points to the center of one figure) and this is the smaller angle size (experimenter pointed to the center of the other figure). Can you show me the bigger angle size?” The six trials were repeated a second time, and children were only asked: “Can you show me the bigger angle size?” All children received feedback regardless of whether or not their responses were correct as was the case in the experimental conditions.

### Procedure

The experimenters were two Chinese female postgraduate students. The pre-test, training, and post-test were all administered individually. The pre-test and post-test sessions lasted 3–5 min each, and the training session lasted 5–7 min. In the pre-test, length-consistent tasks were performed first, then length-neutral tasks, and finally length-inconsistent tasks. In each type of tasks, the tasks of two pairs of acute angles were conducted first, then the tasks of two pairs of obtuse angles, and finally the tasks of two pairs of one acute angle vs. one obtuse angle. In the post-test, only length-inconsistent task was used because children showed perfect or near-perfect performance on length-consistent and length-neutral tasks in the pre-test (see section “Results” for details). The training session was conducted 3 days after the end of the pre-test, and the post-test was conducted 3 days after the training.

## Results

**FIGURE 2:**
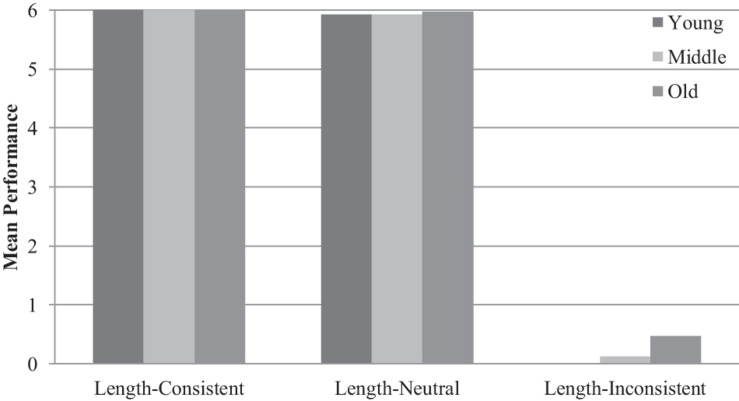
Mean pre-test performance by age group and task type.

In terms of the pre-test results, a 3 (age: young, middle, old) × 2 (gender: boy, girl) × 3 (type: length-consistent, length-neutral, length-inconsistent) mixed-design analysis of variance (ANOVA) revealed a significant main effect of type of task, *F*(1.10, 243.25) = 6,322.87, *p* < 0.001, η*_*p*_*^2^ = 0.97, reflecting children’s better performance at length-consistent tasks and length-neutral tasks than length-inconsistent tasks (see Figure 2 and Table 1). Children’s performance on the former two tasks was near perfect and that on the third task was near zero. In other words, all children had a general sense of the geometric shape of an angle but few were able to separate the dimensions of angle size from the overall size of the angle figure. A significant main effect of age, *F*(2, 222) = 4.42, *p* < 0.05, η*_*p*_*^2^ = 0.04, and a significant Age × Type interaction effect, *F*(2.19, 243.25) = 3.07, *p* < 0.05, η*_*p*_*^2^ = 0.03, were found, reflecting older preschoolers’ better performance on the difficult length-inconsistent task than middle and young preschoolers. There was no significant main effect of gender, *F*(1, 222) = 0.21, *p* = 0.65, η*_*p*_*^2^ = 0.001, nor was there significant interaction effect of Age × Gender × Type, *F*(2.19, 243.25) = 0.69, *p* = 0.52, η*_*p*_*^2^ = 0.01.

**FIGURE 3:**
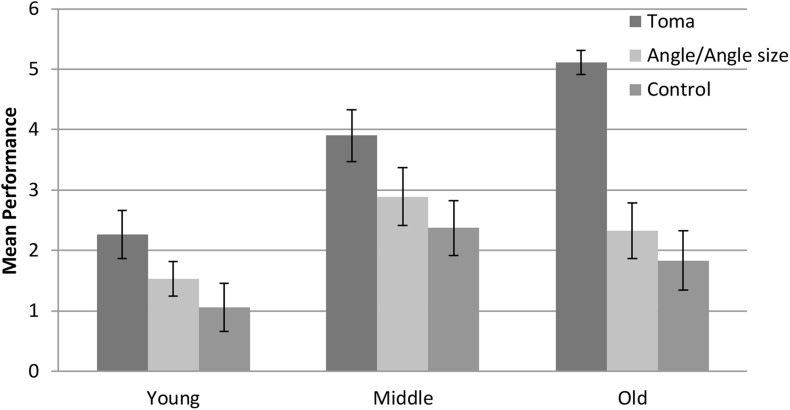
Mean post-test performance on length-inconsistent tasks by condition and age group.

**FIGURE 4:**
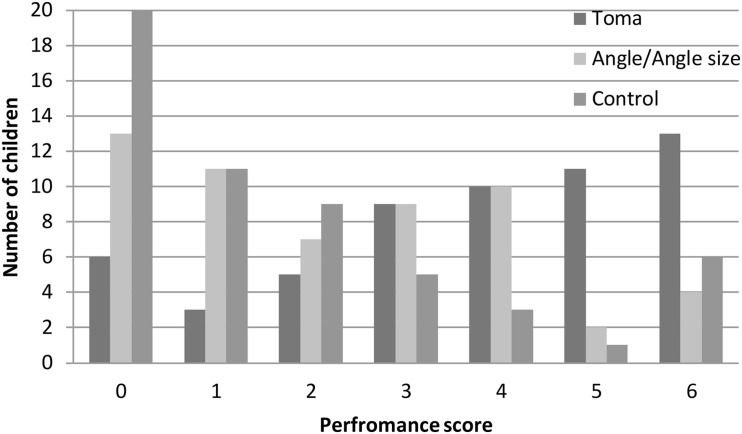
Distribution of children’s post-test scores on the length-inconsistent task by condition.

Given the results of the pre-test (near perfect scores on the length-consistent and length-neutral tasks), only the length-inconsistent task was administered at the post-test. Because the pre-test scores of all children on the length-inconsistent task were 0 points (as an inclusion criterion for the intervention part of the study), we only used the post-test scores as the dependent variable to examine the effect of the intervention. A 3 (age: young, middle, old) × 2 (gender: boy, girl) × 3 (condition: toma, angle/angle size, and control) ANOVA revealed a significant main effect of condition, *F*(2, 150) = 19.25, *p* < 0.001, η*_*p*_*^2^ = 0.20 (see Figure 3). The “toma” group performed significantly better than the other two groups, and the latter two groups did not differ from each other. A more detailed presentation of the condition effect is shown in Figure 4. More children in the “toma” group scored 5 or 6 points than did those in the other two conditions. In contrast, more children in the control and the angle/angle size groups scored 0 or 1 point than did those in the “toma” group. There was a main effect of age, *F*(2, 150) = 13.60, *p* < 0.001, η*_*p*_*^2^ = 0.15, with young preschoolers showing poorer performance than middle and old preschoolers, and the latter two groups not differing from each other. There was not a main effect of gender, *F*(1, 150) = 4.02, *p* = 0.05, η*_*p*_*^2^ = 0.03. Finally, there were no significant interactions between condition and age, *F*(4, 159) = 2.39, *p* = 0.09, η*_*p*_*^2^ = 0.05, between condition and gender, *F*(2, 150) = 0.92, *p* = 0.40, η*_*p*_*^2^ = 0.01, and among condition, age, and gender, *F*(4, 150) = 1.24, *p* = 0.30, η*_*p*_*^2^ = 0.03.

## Discussion

The current study investigated the effectiveness of two interventions on Chinese preschoolers’ understanding of the concept of angle. Results showed that the novel term (toma) intervention based on Gibson et al. (2015) was effective but the “angle/angle size” intervention that is commonly used by Chinese teachers in the classroom was not, suggesting that children’s learning of the concept of angle can be facilitated by two completely different labels (e.g., with a new term), but not by two separate but related labels, for angle and angle size. We further found that Chinese preschoolers showed the same error pattern as their Western partners, but weaker performance on the understanding of angle concept; that gender did not affect the development of the concept of angle and the intervention effect; and that the intervention was equally effective across the age groups included in this study. In the following paragraphs, we compare our results on Chinese children to Gibson et al.’s results on American children and discuss their contributions to our understanding of children’s concepts of angle and angle size and their implications for preschool mathematics education.

First, our pre-test results revealed that Chinese preschoolers showed the same angle misconception and error pattern as American children did (Gibson et al., 2015). Chinese 3–6 year old children showed perfect or near-perfect performance on the length-consistent (mean proportion correct = 100%) and length-neutral tasks (mean proportion correct = 99%) but they almost completely failed on the length-inconsistent tasks (mean proportion correct = 3%, ranging from 0% for young preschoolers to 2% for middle schoolers and to 8% for old preschoolers). Gibson et al. (2015) also found that the length-consistent and length-neutral tasks were easier than the length-inconsistent task, but the accuracy rates were quite different from those found in the current study. The mean proportions of correct responses were 93, 84, and 25% for the three types of tasks, respectively, among their sample of American 4–5 year olds (corresponding to the middle group in our sample). Not surprisingly, in both countries, children seem to have a general sense of the angle figure as an object and can judge its size by one or more of its dimensions (angle size, angle side length, or area), but have difficulty understanding angle size as a separate dimension from the angle figure, as shown in poorer performance when the length of the sides and angle size were inconsistent. This pattern of results is consistent with Van Hiele’s model of the development of geometric reasoning. According to that model, young children use a holistic processing approach to understanding angles and do not focus on separate dimensions such as angle size and side length. It is worth noting that our study did not find gender differences in Chinese preschooler’s angle misconception, which is consistent with some of the previous studies about children’s spatial reasoning (e.g., Spelke et al., 2011), but not others (Spelke, 2005; Davies and Uttal, 2007; Halpern et al., 2007; Tzuriel and Egozi, 2010; Tian et al., 2018). Future research needs to investigate the conditions under which gender differences in children’s geometry cognition and spatial reasoning occur.

Despite the same pattern of task type differences in Gibson et al. (2015) and our studies, the children in our study appeared to perform somewhat better on the length-consistent and length-neutral tasks but much worse on the length-inconsistent tasks as compared to American children in Gibson et al.’s study. Chinese children even younger than the American preschoolers (the young preschooler group in our study) had no difficulty with the length-consistent and neutral tasks. In contrast, Chinese children even older than American preschoolers (the old preschooler group in our study) performed poorly on the length-inconsistent task. In other words, Chinese children’s poorer performance on the length-inconsistent task cannot be attributed to their general sense (or holistic processing) of angle figures. What then would explain their poorer understanding of the concept of angle? Although it is perilous to compare results from different studies, one plausible explanation is that the preschool and kindergarten education guidelines in China emphasize knowledge about numbers, not geometry. The latter is limited to shape naming, recognition, matching, classification, and composition (Department of Basic Education of Ministry of Education of P. R. China. (Ed.)., 2002). Consequently, Chinese parents also pay more attention to mathematics instruction about number cognition such as counting, solving arithmetic problems, and magnitude comparison than to that about geometry (Pan et al., 2006; Zhou et al., 2006, 2007). In contrast, NCTM (1991, 2006) in the United States emphasizes that geometry and spatial reasoning is an important area of mathematic learning for early childhood. Izard and Spelke (2009) and Izard et al. (2014) even found that American 4 year old children were capable of comparing angles across two- and three-dimensional figures. Therefore, cross-country differences in early education practices might have contributed to Chinese preschoolers’ better performance on number cognition as reported in the literature (e.g., Kelly et al., 1999; Zhou et al., 2007; Siegler and Mu, 2008; Xu et al., 2013) but weaker performance on the concept of angle found in this study and possibly other geometric knowledge beyond shape cognition.

Second, our study confirmed that the “toma” intervention was effective among Chinese children as it did for American children. Furthermore, it was equally effective for young, middle, and old preschoolers (as shown by the non-significant interaction between age group and condition). This result is consistent with the whole-object assumption about word-learning bias and the mutual exclusivity bias. According to the whole-object assumption, children tend to interpret a novel term as a label for the whole object and not its parts or properties (e.g., Markman and Hutchinson, 1984; Landau et al., 1988; Hollich et al., 2007). After learning a label for an object, children would have to understand that another label means something else due to the mutual exclusivity bias (Markman and Wachtel, 1988). Specifically, when children learn that “toma” represents the whole angle figure, they would not map “angle” onto the overall angle figure but instead onto a new property, which in the current case is the measure of rotation of an angle. In sum, a novel label helps children to separate the size of an angle from the overall angle figure.

Third, to investigate whether the mere use of two separate labels that refer to the whole figure of an angle and the size of an angle, respectively, would help children overcome the misconception of angle size, we used “angle (jiao)” and “angle size (jiaodu)” in the other experiment condition. These two terms are commonly used by elementary and middle school teachers in China. Results showed that this condition did not improve the learning of the concept of angle. One explanation is that “angle (jiao)” and “angle size (jiaodu)” are synonyms in Chinese vocabulary, so the separate label “angle” is not a novel enough word for Chinese preschoolers to be used to refer to the whole figure of an angle. In other words, the close proximity of the two words and/or the lack of novelty of the new word might have prevented children from separating the whole figure of an angle from the size of an angle.

Our results have important implications for preschool education. Our results suggest that using a novel term as a second label is very effective for teaching young children about angles by helping them attend to angle size as an independent dimension of the overall figure. This recommendation seems to counter the traditional practice of introducing one concept at a time used by Chinese preschool teachers and the common use of angle and angle size distinction by elementary and middle school teachers. Our findings suggest that children can gain a more accurate understanding of a concept by comparing easily confusable meanings with new terms, consistent with the thinking that analogy and structural alignment are powerful learning tools (e.g., Gentner and Markman, 1994, 1997). If Chinese teachers do not want to introduce a novel term and would rather continue using angle and angle size, they should consider using enriching or contextual information to help differentiate the two terms. For example, they can explain that “This is an angle, like a pair of scissors” rather than simply using the ostensive definition (e.g., “This is an angle”). Indeed, previous research has found that different introductory cues produce different learning outcomes (Hall et al., 1993; Congdon et al., 2018).

Finally, we note several limitations of the current study and discuss their implications for future research. First, our pre-test results seemed to show significant differences between Chinese and American preschoolers in their understanding of the concept of angle, but the conclusion needs to be substantiated with a cross-national study using exactly the same experimental and sampling procedure. Second, although we expanded Gibson et al.’s (2015) age range, our study still focused on preschoolers. Given the importance of learning the concept of angle in elementary school, future research should include elementary school students to examine the effectiveness the “toma” intervention and the “angle/angle size” intervention. Perhaps elementary school students may have a better appreciation of the distinction between angle and angle size to benefit from that intervention. Third, as mentioned earlier, our control condition used angle size (jiaodu) to label both angle and angle size. We could have used jiao to represent both. Even though the clearer label of jiaodu did not help children learn the concept of angle, future research nevertheless should consider including an additional control condition using jiao to refer to both the overall figure and angle size. Finally, although we did not find gender differences in this study, future studies, especially those with older children, can also explore demographic and individual differences (such as gender and cognitive abilities) in the effects of interventions.

## Data Availability Statement

The raw data supporting the conclusions of this article will be made available by the authors, without undue reservation, to any qualified researcher.

## Ethics Statement

The studies involving human participants were reviewed and approved by the IRB of Capital Normal University. Written informed consent to participate in this study was provided by the participants’ legal guardian/next of kin.

## Author Contributions

XX had overall responsibility for the research design, data collection, data analysis, and draft writing. CC was responsible for research design, draft editing, as well as interpreting all the data and results. JM, XZ, and MJ were responsible for the data collection and analyzing the data. ZX was responsible for research design, participants’ employment, and reviewing the manuscript. All authors contributed to the article and approved the submitted version.

## Conflict of Interest

The authors declare that the research was conducted in the absence of any commercial or financial relationships that could be construed as a potential conflict of interest.

## References

[B1] Clements D. H. Battista M. T. (1989). Learning of geometric concepts in a Logo environment. *J. Res. Math. Educ.* 20 450–467. 10.2307/749420

[B2] Clements D. H. Battista M. T. (1992). “Geometry and spatial reasoning,” in *Handbook of Research on Mathematics Teaching and Learning*, ed. Grouws D. (New York, NY: Macmillan), 420–464.

[B3] Cohen L. B. Younger B. A. (1984). Infant perception of angular relations. *Infant Behav. Dev.* 7 37–47. 10.1016/S0163-6383(84)80021-1

[B4] Congdon E. L. Vasilyeva M. Mix K. S. Levine S. C. (2018). “From intuitive spatial measurement to understand of units,” in *Visualizing Mathematics. Research in Mathematics Education*, eds Mix K. Battista M. (Cham: Springer), 25–46. 10.1007/978-3-319-98767-5_2

[B5] Davies C. Uttal D. H. (2007). “Map use and the development of spatial cognition,” in *The Emerging Spatial Mind*, eds Plumert J. Spencer J. (New York, NY: Oxford), 219–247. 10.1093/acprof:oso/9780195189223.003.0010

[B6] Department of Basic Education of Ministry of Education of P. R. China. (Ed.). (2002). *Kindergarten Education Guidelines (trial)” Interpretation.* Nanjing, CHN: Jiangsu Education Press. (in Chinese).

[B7] Gentner D. Markman A. B. (1994). Structural alignment in comparison: no difference without similarity. *Psychol. Sci.* 5 152–158. 10.1111/j.1467-9280.1994.tb00652.x

[B8] Gentner D. Markman A. B. (1997). Structure mapping in analogy and similarity. *Am. Psychol.* 52 45–56. 10.1037/0003-066X.52.1.45

[B9] Gibson D. J. Congdon E. L. Levine S. C. (2015). The effects of word-learning bias on children’s concept of angle. *Child Dev.* 86 319–326. 10.1111/cdev.12286 25156505

[B10] Hall D. G. Waxman S. R. Hurwitz W. M. (1993). How two- and four-year-old children interpret adjectives and count nouns. *Child Dev.* 64 1651–1664. 10.2307/1131461

[B11] Halpern D. F. Benbow C. P. Geary D. C. Gur R. C. Hyde J. S. Gernsbacher M. A. (2007). The science of sex differences in science and mathematics. *Psychol. Sci. Public Interest* 8 1–52. 10.1111/j.1529-1006.2007.00032.x 25530726 PMC4270278

[B12] Hollich G. Golinkoff R. M. Hirsh-Pasek K. (2007). Young children associate novel words with complex objects rather than salient parts. *Dev. Psychol.* 43 1051–1061. 10.1037/0012-1649.43.5.1051 17723035

[B13] Huangpu H. (2009). *Understanding of Students of Grade 4-7 about the Angles.* M. A. thesis, East China Normal University, Shanghai.

[B14] Izard V. O’Donnell E. Spelke E. S. (2014). Reading angles in maps. *Child Dev.* 85 237–249. 10.1111/cdev.12114 23647223 PMC3751975

[B15] Izard V. Spelke E. S. (2009). Development of sensitivity to geometry in visual forms. *Hum. Evol.* 23 213–248. 21359132 PMC3045057

[B16] Kelly M. K. Miller K. F. Fang G. Feng G. (1999). When days are numbered: calendar structure and the development of calendar processing in English and Chinese. *J. Exp. Child Psychol.* 73 289–314. 10.1006/jecp.1999.2503 10419645

[B17] Landau B. Smith L. B. Jones S. S. (1988). The importance of shape in early lexical learning. *Cogn. Dev.* 3 299–321. 10.1016/0885-2014(88)90014-7

[B18] Lehrer R. Jenkins M. Osana H. (1998). “Longitudinal study of children’s reasoning about space and geometry,” in *Designing Learning Environments for Developing Understanding of Geometry and Space*, Vol. 1 eds Lehrer R. Chazan D. (Mahwah, NJ: Erlbaum), 137–167.

[B19] Lindquist M. M. Kouba V. L. (1989). “Geometry,” in *Results from the Fourth Mathematics Assessment of the National Assessment of Educational Progress*, ed. Lindquist M. M. (Reston, VA: National Council of Teachers of Mathematics), 44–54.

[B20] Lindskog M. Rogell M. Kenward B. Gredebäck G. (2019). Discrimination of small forms in a deviant-detection paradigm by 10-month-old infants. *Front. Psychol.* 10:1032. 10.3389/fpsyg.2019.01032 31156498 PMC6528582

[B21] Lourenco S. F. Huttenlocher J. (2008). The representation of geometric cues in infancy. *Infancy* 13 103–127. 10.1080/15250000701795572 33412723

[B22] Markman E. M. Hutchinson J. E. (1984). Children’ssensitivity to constraints on word meaning: taxonomic versus thematic relations. *Cogn. Psychol.* 16 1–27. 10.1016/0010-0285(84)90002-1

[B23] Markman E. M. Wachtel G. F. (1988). Children’s use of mutual exclusivity to constrain the meanings of words. *Cogn. Psychol.* 20 121–157. 10.1016/0010-0285(88)90017-5 3365937

[B24] Mitchelmore M. C. White P. (2000). Development of angle concepts by progressive abstraction and generalisation. *Educ. Stud. Math.* 41 209–238. 10.1023/A:1003927811079

[B25] NCTM (1991). *Professional Standards for Teaching Mathematics.* Reston, VA: National Council of Teachers of Mathematics.

[B26] NCTM (2006). *Curriculum Focal Points for Prekindergarten through Grade 8 Mathematics: A Quest for Coherence.* Reston, VA: National Council of Teachers of Mathematics.

[B27] Pan Y. Gauvain M. Liu Z. Cheng L. (2006). American and Chineses parental involvement in young children’s mathematics learning. *Cogn. Dev.* 21 17–35. 10.1016/j.cogdev.2005.08.001

[B28] Schwartz M. Day R. H. Cohen L. B. (1979). Visual shape perception in early infancy. *Monogr. Soc. Res. Child Dev.* 44 1–63. 10.2307/1165963 537616

[B29] Siegler R. S. Mu Y. (2008). Chinese children excel on novel mathematics problems even before elementary school. *Psychol. Sci.* 19 759–763. 10.1111/j.1467-9280.2008.02153.x 18816281

[B30] Slater A. Mattock A. Brown E. Bremner J. G. (1991). Form perception at birth: revisited. *J. Exp. Child Psychol.* 51 395–406. 10.1016/0022-0965(91)90084-6 2072083

[B31] Soja N. N. Carey S. Spelke E. S. (1991). Ontological categories guide young children’s inductions about word meaning: object terms and substance terms. *Cognition* 38 179–211. 10.1016/0010-0277(91)90051-5 2049905

[B32] Spelke E. S. (2005). Sex differences in intrinsic aptitude for mathematics and science: A critical review. *Am. Psychol.* 60 950–958. 10.1037/0003-066x.60.9.950 16366817

[B33] Spelke E. S. Gilmore C. K. McCarthy S. (2011). Kindergarten children’s sensitivity to geometry in maps. *Dev. Sci.* 14 809–821. 10.1111/j.1467-7687.2010.01029.x 21676100 PMC3117203

[B34] Tardif T. Gelman S. A. Xu F. (1999). Putting the “Noun Bias” in context: a comparison of english and mandarin. *Child Dev.* 70 620–635. 10.1111/1467-8624.45

[B35] Tardif T. Shatz M. Naigles L. (1997). Caregiver speech and children’s use of nouns versus verbs: a comparison of English, Italian, and Mandarin. *J. Child Lang.* 24 535–565. 10.1017/S030500099700319x 9519585

[B36] Tian M. Deng Z. Meng Z. Li R. Zhang Z. Qi W. (2018). The impact of individual differences, types of model and social settings on block building performance among chinese preschoolers. *Front. Psychol.* 9:27. 10.3389/fpsyg.2018.00027 29441031 PMC5797599

[B37] Tzuriel D. Egozi G. (2010). Gender differences in spatial ability of young children: the effects of training and processing strategies. *Child Dev.* 81 1417–1430. 10.1111/j.1467-8624.2010.01482.x 20840231

[B38] Van Hiele P. M. (1986). *Structure and Insight. A Theory of Mathematics Education.* London: Academic Press.

[B39] Xu X. Chen C. Pan M. Li N. (2013). Development of numerical estimation in Chinese preschool children. *J. Exp. Child Psychol.* 116 351–366. 10.1016/j.jecp.2013.06.009 23933179

[B40] Zhou X. Chen Y. Chen C. Jiang T. Zhang H. Dong Q. (2007). Chinese kindergartners’ automatic processing of numerical magnitude in stroop-like tasks. *Memery Cogn.* 35 464–470. 10.3758/BF03193286 17691145

[B41] Zhou X. Huang J. Wang Z. Wang B. Zhao Z. Yang L. (2006). Parent - child interaction and children’s number learning. *Early Child Dev. Care* 176 763–775. 10.1080/03004430500232680

